# Gut bacterial profiles in Parkinson's disease: A systematic review

**DOI:** 10.1111/cns.13990

**Published:** 2022-10-25

**Authors:** Zhe Li, Hongfeng Liang, Yingyu Hu, Lin Lu, Chunye Zheng, Yuzhen Fan, Bin Wu, Tao Zou, Xiaodong Luo, Xinchun Zhang, Yan Zeng, Ziyan Liu, Zhicheng Zhou, Zhenyu Yue, Yi Ren, Zhuo Li, Qiaozhen Su, Pingyi Xu

**Affiliations:** ^1^ Department of Neurology The Second Affiliated Hospital of Guangzhou University of Chinese Medicine (Guangdong Provincial Hospital of Chinese Medicine) Guangzhou China; ^2^ Hospital Administration Office Southern Medical University Guangzhou China; ^3^ Department of Neurology The First Affiliated Hospital of Guangzhou Medical University Guangzhou China; ^4^ Genetic Testing Lab The Second Affiliated Hospital of Guangzhou University of Chinese Medicine (Guangdong Provincial Hospital of Chinese Medicine) Guangzhou China; ^5^ Chronic Disease Management Outpatient The Second Affiliated Hospital of Guangzhou University of Chinese Medicine (Guangdong Provincial Hospital of Chinese Medicine) Guangzhou China; ^6^ The Second Clinical College, Guangzhou University of Chinese Medicine Guangzhou China; ^7^ Department of Neurology Friedman Brain Institute, Icahn School of Medicine at Mount Sinai New York New York USA; ^8^ Department of Biomedical Sciences Florida State University College of Medicine Tallahassee Florida USA

**Keywords:** Parkinson's disease, gut bacteria, family, genus, systematic review

## Abstract

**Introduction:**

Recent advances have highlighted the relationships between gut dysbiosis and Parkinson's disease (PD). Microbiota transplantation from PD patients to mice can induce increased alpha‐synuclein‐mediated motor deficits. Human studies have identified differences in the gut microbiota of PD patients compared to healthy controls. We undertook a systematic review to evaluate the available evidence for the involvement of gut bacteria in the etiology of PD.

**Methods:**

The PubMed databank, the China National Knowledge Infrastructure databank, and Wanfang Data were searched from inception until June 2021 to identify human case–control studies that investigated relationships between PD and microbiota quantified from feces. We evaluated the resulting studies focusing on bacterial taxa that were different between PD patients and healthy controls.

**Results:**

Twenty‐six studies were found in which 53 microbial families and 98 genera exhibited differences between patients with PD and healthy controls. The genera identified by more than two studies as increased in PD were *Bifidobacterium*, *Alistipes*, *Christensenella*, *Enterococcus*, *Oscillospira*, *Bilophila*, *Desulfovibrio*, *Escherichia/Shigella*, and *Akkermansia*, while *Prevotella*, *Blautia*, *Faecalibacterium*, *Fusicatenibacter*, and *Haemophilus* had three or more reports of being lower in PD patients. More than one report demonstrated that *Bacteroides*, *Odoribacter*, *Parabacteroides*, *Butyricicoccus*, *Butyrivibrio*, *Clostridium*, *Coprococcus*, *Lachnospira*, *Lactobacillus*, *Megasphaera*, *Phascolarctobacterium*, *Roseburia*, *Ruminococcus*, *Streptococcus*, and *Klebsiella* were altered in both directions.

**Conclusion:**

Our review shows that the involvement of the gut microbiome in the etiology of PD may involve alterations of short‐chain fatty acids (SCFAs)‐producing bacteria and an increase in putative gut pathobionts. SCFAs‐producing bacteria may vary above or below an “optimal range,” causing imbalances. Considering that *Bifidobacterium*, *Lactobacillus*, and *Akkermansia* are beneficial for human health, increased *Bifidobacterium* and *Lactobacillus* in the PD gut microbiome may be associated with PD medications, especially COMT inhibitors, while a high level of *Akkermansia* may be associated with aging.

## Graphical abstract

The role of gut bacteria in association with Parkinson's disease (PD) may involve the alterations of bacteria with the capacity to produce short‐chain fatty acids (SCFAs) and an increase in putative pathobionts, which may work together to potentially impair the intestinal barrier and/or blood–brain barrier integrity, stimulating systemic and neural inflammation. SCFAs‐producing bacteria may reduce or increase outside of an “optimal range”. Considering that *Bifidobacterium*, *Lactobacillus*, and *Akkermansia* are beneficial for human health, the increased *Bifidobacterium* and *Lactobacillus* may be associated with PD medications, especially COMT inhibitors, while a high level of *Akkermansia* may be associated with aging.

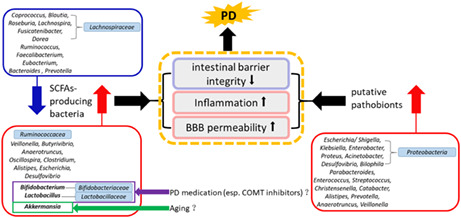

## 1 INTRODUCTION

The gut microbiota refers to the diverse microbial community (bacteria, fungi, archaea, viruses, and protozoa) that colonizes the human gastrointestinal tract.^1^ It is estimated that the number of gut microbiota is up to 10^13^–10^14^ microorganisms, which is significantly more than the number of human cells in the body, and contains more than 100 times as many genes as the human genome.^2^ As it performs many of the functions required for human physiology and survival, it is known as the forgotten organ.^3^ With the development of research, a bidirectional communication system between the gut microbiota and the brain has been recognized, known as the “microbiota‐gut‐brain axis”. Many studies have implicated that gut dysbiosis is closely associated with neurodegenerative diseases, such as Parkinson's disease (PD), Alzheimer's disease, and amyotrophic lateral sclerosis.^4^, ^5^

Parkinson's disease is one of the most common neurodegenerative disorders presenting with motor and non‐motor symptoms. The crucial pathological feature of PD is the aggregation of abnormally folded alpha‐synuclein (α‐syn) proteins, forming intracellular inclusions within the Lewy bodies and Lewy neurites of neurons, which can be found in the central, autonomous, and enteric nervous systems.^6^ Evidence suggests that α‐syn deposition may begin in the neurons of the intestinal submucosa, even up to 8 years before the onset of motor symptoms.^7^, ^8^ The gut microbiota and its products are adjacent to enteric nerves. Alterations in the gut microbiota composition may lead to changes in gut permeability and intestinal barrier function, affecting gastrointestinal epithelial cells and the immune system and the enteric nervous system.^9^ Gut bacteria may activate the immune system through a defective gut barrier, thereby causing a systemic inflammatory response that, in turn, impairs the blood–brain barrier and promotes neuroinflammation and, ultimately, neural injury and degeneration.^4^ Intestinal inflammation triggered by bacterial pathobionts may also contribute to the initiation of α‐syn misfolding,^5^ and further α‐syn pathology propagates in a retrograde manner to the brain via the vagus nerve.^10^ A study of PD model mice confirmed that the gut microbiota contributes to motor deficits and neuroinflammation, and suggested that alterations in the human intestinal microbiome represented a risk factor for PD.^11^ Follow‐up studies have identified characteristic shifts in the gut microbiota associated with the progression of PD, reinforcing the hypothesis that PD develops and is exacerbated due to the altered interplay between gut microbes and the mucosal immune system.^12^, ^13^

Three reports suggested that two genera from the archaea may be involved, while others presented results including only bacterial taxa. Due to different sequencing approaches and statistical methods, the *p* values are not directly comparable. Thus, we systematically reviewed the scientific literature related to human case–control studies concerning gut bacterial composition related to PD. Studies on this topic may provide new insights into the pathogenesis of PD, and allow for innovative therapeutic options such as probiotics,^14^ dietary modification,^15^ and fecal microbiota transplantation.^16^

## 2 MATERIALS AND METHODS

**FIGURE 1:**
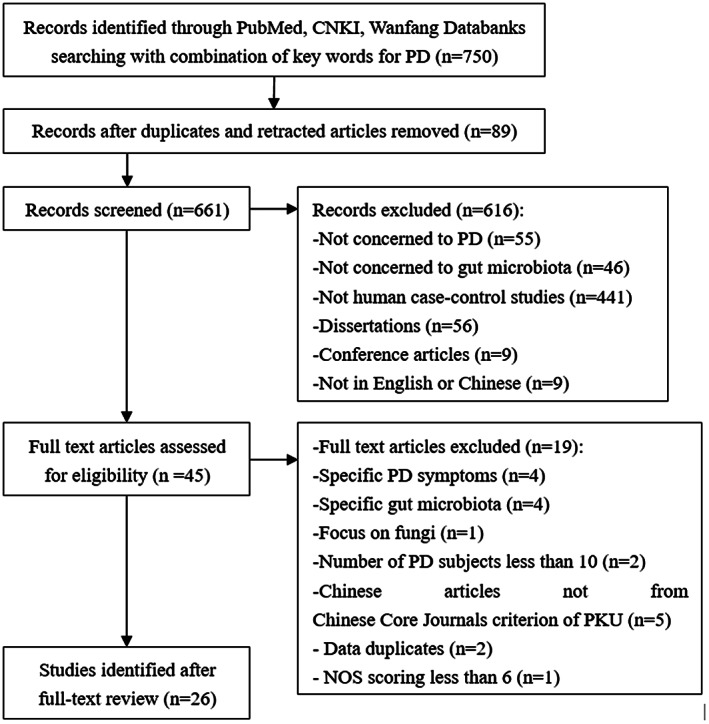
Study flowchart

Two independent reviewers performed systematic electronic literature searches in the PubMed databank, the China National Knowledge Infrastructure (CNKI) databank, and Wanfang Data from inception to June 2021. The following keywords and limits were used: (“Parkinson Disease” OR “Idiopathic Parkinson's Disease” OR “Lewy Body Parkinson's Disease” OR “Parkinson's Disease, Idiopathic” OR “Parkinson's Disease, Lewy Body” OR “Parkinson Disease, Idiopathic” OR “Parkinson's Disease” OR “Idiopathic Parkinson Disease” OR “Lewy Body Parkinson Disease” OR “Primary Parkinsonism” OR “Parkinsonism, Primary” OR “Paralysis Agitans”) AND (“Gastrointestinal Microbiomes” OR “Microbiome, Gastrointestinal” OR “Gut Microbiome” OR “Gut Microbiomes” OR “Microbiome, Gut” OR “Gut Microflora” OR “Microflora, Gut” OR “Gut Microbiota” OR “Gut Microbiotas” OR “Microbiota, Gut” OR “Gastrointestinal Flora” OR “Flora, Gastrointestinal” OR “Gut Flora” OR “Flora, Gut” OR “Gastrointestinal Microbiota” OR “Gastrointestinal Microbiotas” OR “Microbiota, Gastrointestinal” OR “Gastrointestinal Microbial Community” OR “Gastrointestinal Microbial Communities” OR “Microbial Community, Gastrointestinal” OR “Gastrointestinal Microflora” OR “Microflora, Gastrointestinal” OR “Gastric Microbiome” OR “Gastric Microbiomes” OR “Microbiome, Gastric” OR “Intestinal Microbiome” OR “Intestinal Microbiomes” OR “Microbiome, Intestinal” OR “Intestinal Microbiota” OR “Intestinal Microbiotas” OR “Microbiota, Intestinal” OR “Intestinal Microflora” OR “Microflora, Intestinal” OR “Intestinal Flora” OR “Flora, Intestinal” OR “Enteric Bacteria” OR “Bacteria, Enteric”). Original articles were included based on the following criteria: human case–control studies comparing the gut microbiome composition of Parkinson's patients with that of healthy individuals; PD subjects more than 10; use of fecal samples; original microbiome data; written in English or Chinese. The results regarding fungi, archaea, and viruses were excluded because only two genera (*Nitrososphaera*, *Methanobrevibacter*) from archaea were reported in three of the eligible articles,^17^, ^18^, ^19^ and only a single paper focused on fungi without any report of a difference between the PD group and the healthy control (HC) group.^20^ Dissertation and conference articles were excluded. The Newcastle‐Ottawa Scale (NOS) for case–control study^21^ was used to assess quality. A study scoring 6 or higher was deemed to be of sufficient quality. In addition, to guarantee the quality of Chinese articles, we include only those from the Chinese Core Journals criterion of Peking University (PKU). The data were sorted using the Preferred Reporting Items for Systematic Reviews and Meta‐analyses method^22^ (Figure 1).

## 3 RESULTS

### 3.1 Summary of studies included

Following the search strategy, a total of 750 articles were initially retrieved. Abstracts were reviewed manually, and duplicates and retracted articles were removed. Relevant articles were screened from the selected papers, yielding 661 articles. After applying the inclusion and exclusion criteria, 26 studies, including 1963 PD patients and 1639 HCs, were enrolled in the systematic review. Eight originated from China,^17^, ^18^, ^19^, ^23^, ^24^, ^25^, ^26^, ^27^ five from Germany,^28^, ^29^, ^30^, ^31^, ^32^ three from Italy,^33^, ^34^, ^35^ two from the United States,^36^, ^37^ and two from Finland,^38^, ^39^ and the remaining studies were from Japan,^40^ Malaysia,^41^ Canada,^42^ Russia,^43^ Luxembourg^44^ and Australia.^45^ All of the study reports were published within the last 7 years. Studies were assessed for quality using NOS for case–control studies, and the results can be viewed in Table 1.

**TABLE 1:** Characteristics of the included studies

Study	Region	Number of subjects (PD/HC)	Sex % male (PD/HC)	Mean age ± SD (PD/HC)	PD treatment [number (%)]	NOS	Method	Alpha diversity (sample richness: Chao1)	Alpha diversity (sample diversity: Shannon)	Beta diversity (weighted and/or unweighted Unifrac)
Lin et al.^17^	China	74/44	66.2/50.0	60.48 ± 10.72/63.20 ± 6.00	Levodopa 55 (74.3%), COMT inhibitor 4 (5.00%), Dopamine agonist 38 (51.4%), Amantadine 21 (28.4%), DBS 4 (5.00%)	7	Illumina HiSeq sequencing	sd	nd	sd
Qian et al.^19^	China	45/45	48.89/51.11	68.1 ± 8.0/67.9 ± 8.0	Levodopa 39 (86.7%), COMT inhibitor 3 (6.7%), Dopamine agonist 27 (60.0%), MAO‐B inhibitor 9 (20.0%), Anticholinergic 4 (8.9%), Amantadine 7 (15.6%)	8	Illumina MiSeq sequencing	sd (>)	sd (>)	sd*
Zhao et al. 2018^23^	China	24/14	66.67/42.86	73.75 ± 6.26/74.64 ± 5.57	NA	8	Illumina MiSeq sequencing	nd	nd	NA
Li et al.^18^	China	51/48	62.75/39.58	62.4 ± 8.2/62.2 ± 9.2	Carbidopa/levodopa, Dopamine agonist^a^	7	Illumina HiSeq sequencing	sd (<)	sd (>)	sd
Lin et al.^24^	China	80/77	53.6/46.7	64.0 ± 8.8/62.1 ± 4.7	Levodopa 75 (93.4%), COMT inhibitor 15 (18.8%), Dopamine agonist 65 (81.3%), MAO‐B inhibitor 8 (10.0%), Anticholinergic 10 (12.5%), Amantadine 12 (15.0%)	8	Illumina MiSeq sequencing	nd	sd (>)	sd
Qian et al.^25^	China	40/40	47.4/52.5	66.6 ± 7.1/66.3 ± 8.1	Levodopa 35 (87.5%), COMT inhibitor 2 (5.0%), Dopamine agonist 25 (62.5%), MAO‐B inhibitor 10 (25.0%), Benzhexol hydrochloride 5 (12.5%), Amantadine 4 (10.0%)	7	Illumina HiSeq sequencing	NA	sd (>)	sd
Li et al.^26^	China	30/30	66.67/50.00	67 ± 6/65 ± 8	NA	6	Illumina MiSeq sequencing	nd	nd	NA
Zhang et al.^27^	China	63/healthy spouses 63, healthy people 74	63.5/healthy spouses 36.5, healthy people 58.1	64.0 ± 7.4/healthy spouses 65.4 ± 7.4, healthy people 63.4 ± 6.6	Levodopa 54 (85.71%), Dopamine agonist 8 (12.70%), Anticholinergic 5 (7.94%), Amantadine 2 (3.17%)	8	Illumina HiSeq sequencing	NA	NA	nd
Hasegawa et al.^40^	Japan	52/36	40.38/58.33	68.9 ± 6.8/68.4 ± 9.7	Levodopa, COMT inhibitor, Dopamine agonist, MAO‐B inhibitor, Anticholinergic, Amantadine, Zonisamide^a^	7	Yakult Intestinal Flora‐SCAN	NA	NA	NA
Tan et al.^41^	Malaysia	104/96	62.5/37.5	65.4 ± 8.4/62.4 ± 9.0	Levodopa 93 (89.42%), COMT inhibitor 32 (30.77%), Dopamine agonist 59 (56.73%), MAO‐B inhibitor 33 (31.73%), Anticholinergic 15 (14.42%), Amantadine 18 (17.31%)	9	Illumina HiSeq sequencing	nd	nd	NA
Unger et al.^28^	Germany	34/34	70.59/52.94	67.7 ± 8.9/64.6 ± 6.6	Levodopa 24 (70.59%), COMT inhibitor 11 (32.35%), Dopamine agonist 22 (64.71%), MAO‐B inhibitor 21 (61.76%), Anticholinergic 0 (0%), Amantadine 4 (11.76%)	7	96‐well block of the ABI PRISM 7900HT Sequence Detection System	NA	NA	NA
Bedarf et al.^29^	Germany	31/28	100/100	64.8 ± 9.5/65.6 ± 10.4	Levodopa 0 (0%), Dopamine agonist 11 (35.5%), MAO‐B inhibitor 28 (90.3%), Amantadine 26 (89.3%)	6	Illumina HiSeq sequencing	nd	NA	NA
Heintz‐Buschart et al.^30^	Germany	76/78	66/59	68.0 ± 9.7/68.4 ± 6.7	Levodopa 66 (86.8%), COMT inhibitor 4 (5.3%), Dopamine agonist 52 (68.42%), MAO‐B inhibitor 52 (68.42%)	8	Illumina HiSeq sequencing	NA	NA	NA
Weis et al.^31^	Germany	34/25	67.65/44.00	67.88/63.84^b^	Levodopa 24 (70.59%), COMT inhibitor 11 (32.35%), Dopamine agonist 21 (61.76%), MAO‐B inhibitor 21 (61.76%), Amantadine 4 (11.76%), Budipine 1 (2.94%)	7	Illumina MiSeq sequencing	sd (<)	nd	nd
Cosma‐Grigorov et al.^32^	Germany	70/31	54.3/54.8	65.3 ± 10.2/64.3 ± 8.9	Levodopa 50 (71.4%), COMT inhibitor 11 (15.7%), Dopamine agonist 55 (78.6%), MAO‐B inhibitor 44 (62.9%), Amantadine 13 (18.6%)	7	Illumina MiSeq sequencing	nd	nd	sd
Barichella et al.^33^	Italy	193/113	59.6/41.6	67.6 ± 9.7/65.9 ± 9.9	Levodopa 147 (76.2%), COMT inhibitor 29 (15.0%), Dopamine agonist 80 (41.5%), MAO‐B inhibitor 27 (14.0%), Anticholinergic 1 (0.5%), Unmedicated 39 (20.21%)	7	Illumina MiSeq sequencing	sd (>)	sd (>)	sd (>)
Pietrucci et al.^34^	Italy	80/72	65.0/50.0	66.2 ± 8.7/62.6 ± 8.7	Levodopa 66 (82.5%), COMT inhibitor 8 (10.0%), Dopamine agonist 59 (73.8%), MAO‐B inhibitor 58 (72.5%), Anticholinergic 3 (4.0%), Amantadine 3 (4.0%)	7	Illumina MiSeq sequencing	nd	nd	NA
Vascellari et al.^35^	Italy	64/51	68.75/60.78	71.39 ± 10.99/51.67 ± 12.42	Levodopa 64 (100.0%)	7	Illumina MiSeq sequencing	nd	NA	sd
Keshavarzian et al.^36^	USA	38/34	63.16/52.94	61.6 ± 9.4/45.1 ± 14.4	NA	6	Illumina MiSeq sequencing	sd (>)**	sd (>)	NA
Hill‐Burns et al.^37^	USA	197/130	67.0/39.2	68.4 ± 9.2/70.3 ± 8.6	Carbidopa/levodopa 168 (90.8%), COMT inhibitor 37 (20.0%), Dopamine agonist 99 (53.5%), MAO‐B inhibitor 71 (38.4%), Anticholinergic 7 (3.8%), Amantadine 49 (26.5%), Unmedicated 3 (1.6%)	6	Illumina MiSeq sequencing	NA	NA	sd
Scheperjans et al.^38^	Finland	72/72	51.4/50.0	65.3 ± 5.5/64.5 ± 6.9	Levodopa 54.2%, COMT inhibitor 15.3%, Dopamine agonist 77.8%, MAO inhibitor 70.8%, Anticholinergic 8.3%, Unmedicated 2.8%, DBS 2.8%	9	Roche 454 GS FLX Titanium sequencing	nd	nd	sd
Aho et al.^39^	Finland	64/64	51.56/50.00	65.2 ± 5.52/64.45 ± 6.9	Stable patients: Levodopa 22 (53.66%); COMT inhibitors 5 (12.2%); Dopamine agonist^c^ Progressed patients: Levodopa 7 (46.67%); COMT inhibitors 1 (6.67%); Dopamine agonist^c^	7	Illumina MiSeq sequencing	nd	nd	sd
Cirstea et al.^42^	Canada	197/103	61.9/48.5	66 (59–71)/66 (58–71)^d^	Levodopa 180 (91.4%), COMT inhibitor 17 (8.6%), Dopamine agonist 34 (17.3%), MAO‐B inhibitor 33 (16.8%), Amantadine 11 (5.6%)	7	Illumina MiSeq sequencing	nd	nd	sd
Petrov et al.^43^	Russia	89/66	NA	67 (65, 69.75)/63 (61.5, 67.5)^e^	NA	6	Illumina MiSeq sequencing	sd (<)	NA	sd
Baldini et al.^44^	Luxembourg	147/162	68.5/64.2	69.3 ± 8.6/63.3 ± 8.3	Levodopa 98 (66.7%), COMT inhibitor 6 (4.1%), Dopamine agonist 83 (56.5%), MAO‐B inhibitor 61 (41.5%)	8	Illumina MiSeq sequencing	sd (>)	nd	sd
Lubomski et al.^45^	Australia	21/10	52.4/50.0	66.4 ± 9.9/57.3 ± 12.7	DBS cohort: Levodopa 10 (100.0%), COMT inhibitor 4 (40.0%), Dopamine agonist 6 (60.0%), MAO‐B inhibitor 2 (20.0%), Amantadine 1 (10.0%). LCIG cohort: Levodopa 11 (100.0%), COMT inhibitor 5 (45.5%), Dopamine agonist 7 (63.6%), MAO‐B inhibitor 2 (18.2%), Anticholinergic 1 (9.9%), Amantadine 2 (18.2%), Apomorphine 4 (36.4%)	6	Illumina MiSeq sequencing	NA	nd	sd

*Note*: <, a lower abundance in PD patients when compared to HCs; >, a higher abundance in PD patients when compared to HCs.

Abbreviations: COMT, catechol‐O‐methyltransferase; DBS, deep brain stimulation; HC, healthy control; LCIG, levodopa–carbidopa intestinal gel; MAO‐B, monoamine oxidase‐B; NA, not available; nd, no difference; NOS, Newcastle‐Ottawa Scale; PD, Parkinson's disease; sd, significant difference; SD, standard deviation.

^a^ Number and percentage of subjects using PD medication was not given.

^b^ SD was not given.

^c^ Number and percentage of subjects using dopamine agonist was not given.

^d^ Data shown in median (interquartile range).

^e^ Data shown in mean (95% confidential interval).

* Significant difference shown in unweighted UniFrac but not in weighted UniFrac.

** Significant difference shown in richness (Margalef).

Sixteen studies used the Illumina MiSeq sequencing method, while seven used the Illumina HiSeq sequencing method. One study used the Roche 454 GS FLX Titanium sequencing method. Two studies used other taxonomic identification and quantification methods: Yakult Intestinal Flora‐SCAN or 96‐well block of the ABI PRISM 7900HT Sequence Detection System. The Illumina MiSeq and Illumina HiSeq sequencing methods assess the whole microbiome and may detect surprising unknown species, whereas the last two methods only assess targeted microbial taxa and are used to reproduce previously reported findings.^46^

### 3.2 Diversity changes in gut bacteria in PD patients

The gut microbial ecosystem can be defined by its alpha and beta diversity, with alpha diversity based on the number of individual species detected, and beta diversity assessing how different the gut microbiome composition is between subjects.^47^ The Chao1 index and Shannon index represent sample richness and diversity, respectively, at the level of alpha diversity. Eight studies showed significant differences in sample richness between PD cases and HCs, while 11 studies reported no significant difference between the two groups. Six studies displayed significantly higher diversity in the PD group than in the HC group, while 12 studies reported no significant difference between the two groups. However, in a majority of the studies that reported information on overall beta diversity, significant differences were found between PD and HC (Table 1).

### 3.3 Alterations in the fecal bacteria composition of PD patients

#### 3.3.1 Family

**FIGURE 2:**
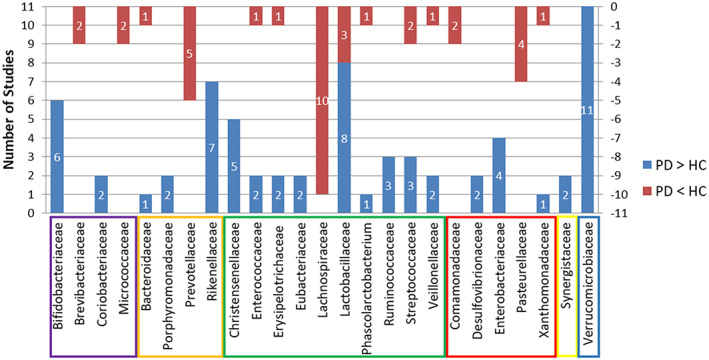
The number of studies showing differences between the PD and HC groups in the top 25 families of fecal bacteria. The blue bar represents the number of studies showing a higher abundance in PD patients than in HCs. The red bar represents the number of studies showing a lower abundance in PD patients than in HCs. Family members of *Actinobacteria* are indicated in a purple frame, *Bacteroidetes* are indicated in an orange frame, *Firmicutes* are indicated in a green frame, *Proteobacteria* are indicated in a red frame, member of *Synergistetes* is indicated in a yellow frame and *Verrucomicrobia* is indicated in a blue frame. HC, healthy control; PD, Parkinson's disease

On the family level, five and three studies, respectively, reported higher abundance in PD patients than in HCs of the *Christensenellaceae* and *Ruminococcaceae* families, which are under the *Clostridiales* order. However, 10 studies reported that the *Lachnospiraceae* under *Clostridiales* exhibited lower abundance in the PD group than in the HC group. *Lactobacillaceae* from *Lactobacillales* was found to be significantly different between the two groups in 11 studies, of which eight showed higher abundance while the other three showed lower abundance in the PD group. Under the *Bacteroidales* order, *Prevotellaceae* showed decreased levels in PD compared to HC in five studies, while more *Rikenellaceae* were detected in PD groups in seven studies. *Bifidobacteriaceae* were more abundant in PD patients in six studies. All 11 studies demonstrated that *Verrucomicrobiaceae* was more abundant in PD patients than in HCs. Other families identified by more than one report as elevated in PD compared to HC included *Coriobacteriaceae*, *Porphyromonadaceae*, *Eubacteriaceae*, *Desulfovibrionaceae*, *Enterobacteriaceae*, and *Synergistaceae*. *Brevibacteriaceae*, *Micrococcaceae*, *Comamonadaceae* and *Pasteurellaceae* were reported in two or more studies as being lower in PD patients than in HCs. More than one report demonstrated that *Bacteroidaceae*, *Enterococcaceae*, *Erysipelotrichaceae*, *Phascolarctobacterium*, *Streptococcaceae*, *Veillonellaceae*, and *Xanthomonadaceae* were altered in both directions. See Figure 2 and Table 2.

**TABLE 2:** Taxonomic changes in family

Phylum	Class	Order	Family	PD > HC	PD < HC
*Firmicutes*	*Negativicutes*	*Acidaminococcales*	*Acidaminococcaceae*	[18]	
		*Veillonellales*	*Veillonellaceae*	[18, 23]	[45]
	*Clostridia*	*Clostridiales*	*Christensenellaceae*	[26, 33, 37, 41, 42]	
			*Lachnospiraceae*		[17, 29, 32, 33, 34, 35, 36, 37, 39, 42]
			*Ruminococcaceae*	[18, 26, 38]	
			*Clostridiaceae*	[36]	
		*Eubacteriales*	*Eubacteriaceae*	[17, 35]	
			*Catabacteriaceae*	[41]	
			*Peptostreptococcaceae*	[26]	
	*Tissierellia*	*Tissierellales*	*Tissierellaceae*	[37]	
	*Bacilli*	*Lactobacillales*	*Enterococcaceae*	[24, 34]	[28]
			*Streptococcaceae*	[23, 25, 35]	[17, 29]
			*Lactobacillaceae*	[23, 24, 25, 33, 34, 37, 38, 41]	[18, 19, 28]
			*Carnobacteriaceae*	[25]	
			*Aerococcaceae*	[17]	
		*Gemellales*	*Gemellaceae*		[17]
	*Erysipelotrichia*	*Erysipelotrichales*	*Coprobacillaceae*		[36]
			*Erysipelotrichaceae*	[19, 24]	[29]
*Bacteroidetes*	*Bacteroidia*	*Bacteroidales*	*Bacteroidaceae*	[36]	[35]
			*Rikenellaceae*	[18, 19, 24, 25, 26, 35, 39]	
			*Prevotellaceae*		[24, 26, 29, 38, 39]
			*Porphyromonadaceae*	[18, 24]	
			*Odoribacteraceae*	[24]	
	*Chitinophagia*	*Chitinophagales*	*Chitinophagaceae*	[19]	
	*Flavobacteriia*	*Flavobacteriales*	*Flavobacteriaceae*		[29]
	*Sphingobacteriia*	Sphingobacteriales	*Sphingobacteriaceae*		[35]
*Actinobacteria*	*Actinomycetia*	*Actinomycetales*	*Actinomycetaceae*		[17]
		*Micrococcales*	*Micrococcaceae*		[17, 29]
			*Microbacteriaceae*	[35]	
			*Brevibacteriaceae*		[17, 35]
			*Intrasporangiaceae*	[17]	
		*Bifidobacteriales*	*Bifidobacteriaceae*	[17, 23, 33, 35, 37, 39]	
	*Coriobacteriia*	*Coriobacteriales*	*Coriobacteriaceae*	[33, 35]	
*Proteobacteria*	*Gammaproteobacteria*	*Pasteurellales*	*Pasteurellaceae*		[17, 23, 37, 45]
		*Enterobacteriales*	*Enterobacteriaceae*	[23, 28, 33, 34]	
		*Xanthomonadales*	*Xanthomonadaceae*	[19]	[17]
		*Pseudomonadales*	*Moraxellaceae*	[19	
		*Oceanospirillales*	*Halmonadaceae*		[17]
		*Alteromonadales*	*Idiomarinaceae*		[17]
	*Alphaproteobacteria*	*Rhizobiales*	*Bradyrhizobiaceae*	[38]	
		*Sphingomonadales*	*Sphingomonadaceae*	[19]	[17]
		*Hyphomicrobiales*	*Methylobacteriaceae*		[17]
			*Brucellaceae*		[17]
		*Hyphomonadales*	*Hyphomonadaceae*		[17]
	*Betaproteobacteria*	*Burkholderiales*	*Alcaligenaceae*		[35]
			*Comamonadaceae*		[17, 35]
			*Sutterellaceae*	[32]	
	*Deltaproteobacteria*	*Desulfovibrionales*	*Desulfohalobiaceae*	[35]	
			*Desulfovibrionaceae*	[17, 42]	
*Verrucomicrobia*	*Verrucomicrobiae*	*Verrucomicrobiales*	*Verrucomicrobiaceae*	[18, 24, 29, 30, 33, 35, 36, 37, 38, 41, 45]	
	*Opitutae*	*Puniceicoccales*	*Puniceicoccaceae*		[39]
*Deferribacteres*	*Deferribacteres*	*Deferribacterales*	*Deferribacteraceae*	[24]	
*Synergistetes*	*Synergistia*	*Synergistales*	*Synergistaceae*	[25, 41]	

Abbreviations: HC, healthy control; PD, Parkinson's disease.

#### 3.3.2 Genus

**FIGURE 3:**
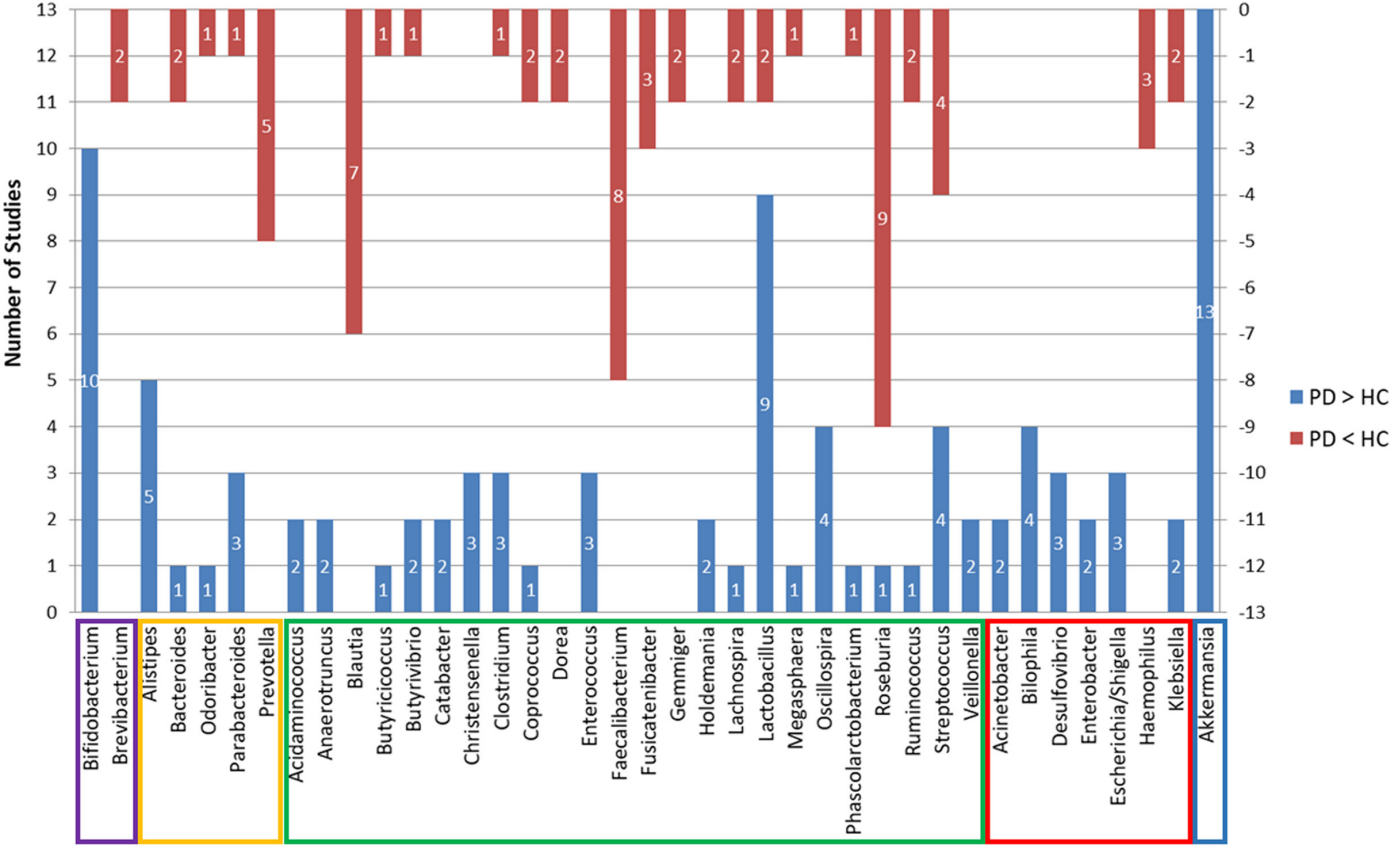
The number of studies showing differences between the PD and HC groups in the top 39 genera of fecal bacteria. The blue bar represents the number of studies showing a higher abundance in PD patients than in HCs. The red bar represents the number of studies showing a lower abundance in PD patients than in HCs. Genus members of *Actinobacteria* are indicated in a purple frame, *Bacteroidetes* are indicated in an orange frame, *Firmicutes* are indicated in a green frame, *Proteobacteria* are indicated in a red frame, and member of *Verrucomicrobia* is indicated in a blue frame. HC, healthy control; PD, Parkinson's disease

All studies identified taxa at the genus level finding 98 genera that distinguished the diagnostic groups as follows: 46 genera were more abundant in PD, 37 were less abundant, and 15 studies had variable findings. The genera identified by more than two studies as increased in PD were *Bifidobacterium*, *Alistipes*, *Christensenella*, *Enterococcus*, *Oscillospira*, *Bilophila*, *Desulfovibrio*, *Escherichia/Shigella*, and *Akkermansia*, while *Prevotella*, *Blautia*, *Faecalibacterium*, *Fusicatenibacter*, and *Haemophilus* had three or more reports of being lower in PD patients. More than one report demonstrated that *Bacteroides*, *Odoribacter*, *Parabacteroides*, *Butyricicoccus*, *Butyrivibrio*, *Clostridium*, *Coprococcus*, *Lachnospira*, *Lactobacillus*, Megasphaera, *Phascolarctobacterium*, *Roseburia*, *Ruminococcus*, *Streptococcus* and *Klebsiella* were altered in both directions. See Figure 3 and Table 3.

**TABLE 3:** Taxonomic changes in genus

Phylum	Class	Order	Family	Genus	PD > HC	PD < HC
*Firmicutes*	*Negativicutes*	*Acidaminococcales*	*Acidaminococcaceae*	*Acidaminococcus*	[23, 44]	
				*Phascolarctobacterium*	[18]	[29]
		*Selenomonadales*	*Selenomonadaceae*	*Megamonas*	[23]	
				*Mitsuokella*		[23]
		*Veillonellales*	*Veillonellaceae*	*Megasphaera*		[23]
				*Dialister*	[17]	
				*Veillonella*	[24, 35]	
	*Clostridia*	*Eubacteriales*	*Christensenellaceae*	*Christensenella*	[41, 43, 44]	
				*Catabacter*	[41, 43]	
			*Lachnospiraceae*	*Coprococcus*	[18]	[35, 36]
				*Blautia*		[17, 23, 35, 36, 37, 39, 45]
				*Roseburia*	[18]	[17, 33, 34, 35, 36, 37, 39, 42, 45]
				*Lachnospira*	[18]	[17, 35]
				*Anaerostipes*	[35]	
				*Fusicatenibacter*		[31, 32, 45]
				*Dorea*		[36, 43]
				*Butyrivibrio*	[26, 41]	[35]
				*Pseudobutyrivibrio*		[35]
				*Lachnobacterium*	[17]	
			*Oscillospiraceae*	*Ruminococcus*	[18]	[23, 33]
				*Faecalibacterium*		[17, 23, 24, 31, 32, 37, 43, 45]
				*Anaerotruncus*	[30, 44]	
				*Oscillospira*	[27, 33, 36, 43]	
				*Gemmiger*		[32, 45]
			*Clostridiaceae*	*Clostridium*	[19, 30, 35]	[39]
				*Butyricicoccus*	[19]	[45]
			*Peptostreptococcaceae*	*Peptoclostridium*	[26]	
			*Eubacteriaceae*	*Eubacterium*		[29]
				*Acetobacterium*	[35]	
		*Thermoanaerobacterales*	*Thermoanaerobacterales Family III*	*Caldicellulosiruptor*	[35]	
	*Bacilli*	*Lactobacillales*	*Enterococcaceae*	*Enterococcus*	[23, 24, 34]	
			*Streptococcaceae*	*Streptococcus*	[23, 25, 35, 44]	[17, 18, 29, 45]
			*Lactobacillaceae*	*Lactobacillus*	[24, 25, 26, 34, 37, 40, 41, 43, 44]	[18, 19]
			*Carnobacteriaceae*	*Granulicatella*	[25]	
				*Alloiococcus*	[17]	
	*Erysipelotrichia*	*Erysipelotrichales*	*Erysipelotrichaceae*	*Holdemania*	[17, 25]	
				*Bulleidia*		[17]
				*Allobaculum*		[17]
				*Erysipelatoclostridium*	[26]	
			*Turicibacteraceae*	*Turicibacter*	[44]	
	*Tissierellia*	*Tissierellales*	*Peptoniphilaceae*	*Peptoniphilus*	[31]	
*Bacteroidetes*	*Bacteroidia*	*Bacteroidales*	*Bacteroidaceae*	*Bacteroides*	[36]	[35, 43]
			*Rikenellaceae*	*Alistipes*	[17, 18, 19, 25, 26]	
			*Prevotellaceae*	*Prevotella*		[24, 26, 29, 39, 43]
				*Paraprevotella*	[19]	
			*Tannerellaceae*	*Parabacteroides*	[18, 24, 33]	[35]
			*Odoribacteraceae*	*Butyricimonas*	[24]	
				*Odoribacter*	[24]	[35]
	*Chitinophagia*	*Chitinophagales*	*Chitinophagaceae*	*Sediminibacterium*		[19]
	*Flavobacteriia*	*Flavobacteriales*	*Flavobacteriaceae*	*Capnocytophaga*		[29]
	*Sphingobacteriia*	*Sphingobacteriales*	*Sphingobacteriaceae*	*Pedobacter*		[17]
				*Sphingobacterium*		[17]
*Actinobacteria*	*Actinomycetia*	*Actinomycetales*	*Actinomycetaceae*	*Actinomyces*		[17]
		*Micrococcales*	*Micrococcaceae*	*Rothia*		[29]
			*Brevibacteriaceae*	*Brevibacterium*		[17, 35]
			*Intrasporangiaceae*	*Knoellia*		[17]
		*Bifidobacteriales*	*Bifidobacteriaceae*	*Bifidobacterium*	[17, 26, 28, 33, 35, 37, 39, 41, 42, 43]	
				*Gardnerella*	[17]	
		*Corynebacteriales*	*Dietziaceae*	*Dietzia*		
	*Coriobacteriia*	*Coriobacteriales*	*Coriobacteriaceae*	*Collinsella*	[42]	
			*Atopobiaceae*	*Atopobium*		[29]
		*Eggerthellales*	*Eggerthellaceae*	*Gordonibacter*	[25]	
				*Slackia*	[35]	
*Proteobacteria*	*Gammaproteobacteria*	*Pasteurellales*	*Pasteurellaceae*	*Haemophilus*		[17, 23, 45]
		*Enterobacteriales*	*Enterobacteriaceae*	*Escherichia/Shigella*	[23, 34, 35]	
				*Enterobacter*	[25, 35]	
				*Klebsiella*	[19, 34]	[29, 35]
				*Proteus*	[23]	
				*Citrobacter*	[34]	
				*Salmonella*	[34]	
		*Enterobacterales*	*Morganellaceae*	*Morganella*	[17]	
			*Yersiniaceae*	*Serratia*	[35]	
		*Pseudomonadales*	*Moraxellaceae*	*Acinetobacter*	[19, 23]	
		*Aeromonadales*	*Succinivibrionaceae*	*Succinatimonas*		[17]
		*Oceanospirillales*	*Halomonadaceae*	*Halomonas*		[17]
		*Alteromonadales*	*Idiomarinaceae*	*Pseudidiomarina*		[17]
		*Xanthomonadales*	*Xanthomonadaceae*	*Stenotrophomonas*		[17]
	*Betaproteobacteria*	*Burkholderiales*	*Sutterellaceae*	*Sutterella*		[35]
			*Comamonadaceae*	*Aquabacterium*	[19]	
			*Burkholderiaceae*	*Ralstonia*	[17]	
		*Neisseriales*	*Neisseriaceae*	*Eikenella*		[17]
	*Alphaproteobacteria*	*Sphingomonadales*	*Sphingomonadaceae*	*Sphingomonas*	[19]	
		*Hyphomicrobiales*	*Brucellaceae*	*Ochrobactrum*		[17]
				*Pseudochrobactrum*		[17]
				*Mycoplana*		[17]
			*Methylobacteriaceae*	*Methylobacterium*		[17]
		*Rhodobacterales*	*Roseobacteraceae*	*Rubellimicrobium*		[17]
		*Hyphomonadales*	*Hyphomonadaceae*	*Hyphomonas*		[17]
		*Caulobacterales*	*Caulobacteraceae*	*Brevundimonas*		[17]
	*Deltaproteobacteria*	*Desulfovibrionales*	*Desulfovibrionaceae*	*Bilophila*	[17, 24, 42, 44]	
				*Desulfovibrio*	[17, 19, 35]	
			*Desulfohalobiaceae*	*Desulfonauticus*	[35]	
*Verrucomicrobia*	*Verrucomicrobiae*	*Verrucomicrobiales*	*Akkermansiaceae*	*Akkermansia*	[18, 24, 26, 27, 29, 30, 33, 35, 36, 37, 41, 44, 45]	
				*Prosthecobacter*	[35]	
*Fusobacteria*	*Fusobacteria*	*Fusobacteriales*	*Fusobacteriaceae*	*Fusobacterium*		[27]
*Deferribacteres*	*Deferribacteres*	*Deferribacterales*	*Deferribacteraceae*	*Mucispirillum*	[24]	
*Cyanobacteria*	Cyanophyceae	*Nostocales*	*Aphanizomenonaceae*	*Dolichospermum*		[35]
*Synergistetes*	*Synergistia*	*Synergistales*	*Synergistaceae*	*Cloacibacillus*	[41]	

Abbreviations: HC, healthy control; PD, Parkinson's disease.

## 4 DISCUSSION

All studies reviewed found significant differences in the family and genus levels between the PD and HC groups. Some studies showed contradictory results regarding either microbial diversity, relative abundance, or directionality of differences in taxa associated with PD, which may be attributed to the study population, sample collection, laboratory procedures, and sequencing methodological inconsistencies.^48^ Nevertheless, several studies suggest that there may be specific associations between gut particular microbial profiles and PD. In total, 98 bacterial genera were reported from 26 studies; however, only 39 bacterial genera showed a significant increase or decrease in more than one study. Therefore, we put emphasis on discussing these 39 more or less abundant bacterial genera and their parental families in this review.

Gut bacteria are commensals that earn their energy via fermentation of the host's dietary intake and human secretions, such as mucin, to produce short‐chain fatty acids (SCFAs) (butyrate, propionate, and acetate).^49^ We observed that some of the bacteria that have the capacity to produce SCFAs, such as *Lachnospiraceae* (*Coprococcus*, *Blautia*, *Roseburia*, *Lachnospira*, *Fusicatenibacter*, and *Dorea*), *Ruminococcus*, *Faecalibacterium*, *Eubacterium*, *Bacteroides* and *Prevotella*,^49^, ^50^, ^51^ showed a decrease in the PD group microbiome compared to HC in most of the studies. Simultaneously, we found that some of the SCFAs‐producing bacteria, such as *Ruminococcaceae*, *Veillonella*, *Butyrivibrio*, *Anaerotruncus*, *Oscillospira*, *Clostridium*, *Lactobacillus*, *Alistipes*, *Bifidobacterium*, *Escherichia*, *Desulfovibrio* and *Akkermansia*,^49^, ^50^, ^51^ were increased in PD patients. SCFAs are vital for intestinal barrier integrity, and can also influence the enteric nervous system, stimulate systemic anti‐inflammatory properties, promote normal microglial development, and potentially affect epigenesis in the central nervous system.^52^ SCFAs are modulated by different genes and enzymes exhibited by various bacteria, which are involved in particular synthesis pathways.^53^, ^54^, ^55^ As suggested by accumulating clinical evidence, lower levels of fecal SCFAs occur in patients with PD than controls,^28^, ^41^, ^56^, ^57^ so we infer that an alteration of SCFAs‐producing bacteria in PD patients collectively modifies their genetic potential to produce enzymes needed for SCFAs formation,^49^ leading to the depletion of SCFAs in the gut. Four pathways participate in the formation of butyrate: the acetyl coenzyme A (CoA) pathway (Ac pathway) fueled by carbohydrates and the glutarate, 4‐aminobutyrate, and lysine pathways fed by proteins.^53^, ^54^ Bacteria exhibiting the Ac pathway are arranged according to their terminal enzymes butyryl‐CoA:acetate CoA transferase (*but*) and butyrate kinase (*buk*).^53^ Three pathways participate in the formation of propionate: the acrylate pathway detecting the lcdA gene and encoding lactoyl‐CoA dehydratase, the propanediol pathway based on the pduP gene encoding propionaldehyde dehydrogenase, and the succinate pathway based on the mmdA gene encoding methylmalonyl‐CoA decarboxylase.^54^, ^55^ In type 2 diabetes patients, mean abundances of the Ac pathway were reduced compared with HCs, in particular, due to a decline in bacteria containing *but* and *buk* (several *Lachnospiraceae* and/or a few *Ruminococcaceae*), whereas bacteria lacking both *but* and *buk* enzymes and protein‐fed pathways were more abundant in type 2 diabetes samples (such as *Oscillibacter* and *Pseudoflavonifractor*).^53^ In colorectal cancer patients, several *Lachnospiraceae* displayed lower levels in patient samples, which was balanced by an increase in *Ruminococcaceae*. Bacteria exhibiting protein‐fed pathways were also elevated in colorectal cancer patients.^53^

Numerous putative pathobionts were significantly altered in the fecal samples of PD patients, which is another important characteristic. In Figure 3, all the genera reported under the phylum *Proteobacteria* are putative pathobionts. *Escherichia* and *Shigella* are among the leading causes of diarrhea^58^ and produce Shiga toxin and lipopolysaccharide, which can damage the striatal microvasculature and astrocytes causing motor deficits, increasing blood–brain barrier permeability, and leading to neuronal degeneration.^59^ *Klebsiella* is a natural inhabitant of the gastrointestinal tract microbiome of healthy humans and animals, but it often causes extraintestinal infections, including urinary tract infections, pneumoniae, pyogenic liver abscesses, septicemia,^60^ and even central nervous system infections.^61^, ^62^ *Enterobacter* spp. is the third most common human pathogen, after *Escherichia coli* and *Klebsiella pneumoniae*, and is involved in a variety of infections, such as bloodstream and intra‐abdominal infections.^63^ *Proteus* is recognized clinically as a cause of urinary tract infections and gastrointestinal conditions, such as gastroenteritis (spontaneous and foodborne), appendicitis, colonization of devices such as nasogastric tubes, and Crohn's disease.^64^ It has been demonstrated that *Proteus mirabilis*, isolated from PD mice, was able to significantly induce motor deficits, and additionally can cause dopaminergic neuronal damage and inflammation, as well as α‐syn aggregation in the brain and colon, suggesting a role of *P. mirabilis* in PD pathogenesis in the brain.^65^ *Acinetobacter* commonly causes nosocomial infections, predominantly aspiration pneumonia, and catheter‐associated bacteremia, but can also cause soft tissue and urinary tract infections.^66^ *Haemophilus* can cause a variety of mild and severe infections, including bacteremia, meningitis, pneumonia, sinusitis, otitis media, cellulitis, and epiglottitis. *Desulfovibrio* is a group of gram‐negative, motile, sulfate‐reducing, anaerobic bacteria, and one of its four species, *Desulfovibrio desulfuricans*, can cause human infections on rare occasions, mainly bacteremia and intra‐abdominal infections.^67^ *Bilophila* is thought to be virulent since it is the third most common anaerobic isolate in studies of gangrenous and perforated appendicitis.^68^ One study showed that the microbiota of ICU patients with sepsis has an increased abundance of microbes tightly associated with inflammation, such as *Bilophila* and *Parabacteroides* species.^69^ *Parabacteroides* and *Bacteroides* under the phylum *Bacteroidetes* are generally opportunistic pathogens in infectious diseases and are able to develop antimicrobial drug resistance.^70^ Regarding the phylum *Firmicutes*, *Enterococcus* is one of the most common causes of hospital‐associated infections, especially *Enterococcus faecalis* and *Enterococcus faecium*, which can produce extracellular superoxide and hydrogen peroxide that damage colonic epithelial cell DNA.^50^, ^71^ A report showed that the abundance of pathogenic species, such as *Enterococcus* spp., was differentially increased in ICU sepsis patients who died.^69^ *Streptococcus* can cause mild human infections such as pharyngitis and impetigo, as well as serious infections such as necrotizing fasciitis and streptococcal toxic shock syndrome. Furthermore, repeated *Streptococcus* infections may trigger autoimmune diseases.^72^ *Christensenella* is Gram‐negative, strictly anaerobic, non‐sporeforming, non‐motile, short rods, and its type species is *Christensenella minuta*.^73^ *Christensenella minuta* has been reported to be a potential human pathogen, isolated in a mixed infection with *Desulfovibrio desulfuricans* from an acute appendicitis patient.^67^ *Catabacter* and *Christensenella* belong to the same family *Christensenellaceae*. One of its species, *Catabacter hongkongensis*, is a strictly anaerobic, catalase‐positive, motile, non‐sporulating, gram‐positive coccobacillus that mostly presents with gastrointestinal or biliary tract infections associated with a poor prognosis.^74^, ^75^ Other bacteria in Figure 3 that could cause infection in humans, such as *Brevibacterium*, *Alistipes*, *Prevotella*, *Anaerotruncus*, and *Veillonella*, are rare.^76^, ^77^, ^78^, ^79^, ^80^, ^81^ In our study, most of the abovementioned bacteria in this paragraph were significantly increased in the PD group compared to HC in the included studies, except *Brevibacterium*, *Bacteroides*, and *Haemophilus*. Evidence suggests that there are systemic immuno‐inflammatory processes in PD that could be related to an abnormal increase in putative pathobionts. These putative pathobionts may produce endotoxins and neurotoxins that elevate inflammation and potentially lead to an environment well suited for the emergence and development of PD pathology.^50^

The increase in *Bifidobacterium* and *Bifidobacteriaceae* in PD patients' gut microbiome was consistent across all reported studies, and the increase in *Lactobacillus* and *Lactobacillaceae* in PD fecal samples was also reproduced in most of the reported studies. However, in PD patients' 2‐year follow‐up studies, *Bifidobacteriaceae* and *Bifidobacterium*, as well as *Lactobacillaceae* and *Lactobacillus* gradually decreased over time,^12^, ^39^ especially in the stable patient group.^12^ The fecal *Bifidobacterium* counts were even lower in the deteriorated group than in the stable group at baseline.^12^ This implies that *Bifidobacterium* and *Lactobacillus* rapidly decrease in patients with accelerated PD pathology.^12^ In daily life, *Bifidobacterium* and *Lactobacillus* are commonly considered to be beneficial bacteria. Several clinical studies have attested that administration of probiotics (mostly *Bifidobacterium* and *Lactobacillus*) could improve the symptoms associated with constipation and even motor function in PD patients.^14^ The reason that abundance of *Bifidobacteriaceae* and *Bifidobacterium*, as well as *Lactobacillaceae* and *Lactobacillus* are higher in PD patients than HCs may come down to two points. In the foregoing paragraph, we discussed *Bifidobacterium* and *Lactobacillus* as SCFAs‐producing bacteria. In our earlier study, increased *Bifidobacteriaceae* and *Bifidobacterium*, as well as *Lactobacillaceae* and *Lactobacillus*, were found to have significant correlations with clinical inflammatory indicators including neutrophil percentage, monocyte percentage, and monocyte count.^82^ On the other hand, PD medication, especially catechol‐O‐methyltransferase (COMT) inhibitors, may have an effect on them. Barichella et al.^33^ noticed that the use of COMT inhibitors elevated the level of *Lactobacillaceae*. Hill‐Burns et al.^37^ excluded patients who were on COMT inhibitors or anticholinergics and found a remarkable reduction in the association signal for *Bifidobacterium* at the OTU level. Weis et al.^31^ discovered that PD patients treated with entacapone showed significantly higher relative abundances of *Bifidobacteriaceae* and *Bifidobacterium* than controls. Whether the increased abundance of *Bifidobacterium* and *Lactobacillus* is related to the proinflammatory gut environment or is affected by PD medication remains to be demonstrated.

Additionally, a higher abundance of the genus *Akkermansia* in the gut microbiome of PD patients was consistent in all 13 reported studies. To date, only two recognized species of the *Akkermansia* genus have been described,^83^ and *Akkermansia muciniphila* is the sole species found in human stool.^84^ *Akkermansia muciniphila*, a mucin‐degrading bacterium,^84^ is abundant in the mucus layer of the digestive tract^83^ and exerts a positive modulation of mucus thickness and gut barrier integrity.^85^ *Akkermansia muciniphila* stimulates the mucus turnover rate by making SCFAs from the degraded mucin to promote mucus thickness, the preferable energy source for the host epithelium, which synthesizes and secretes mucin.^85^ In addition to promoting gut barrier integrity, *A. muciniphila* could modulate the immune response, inhibit inflammation, and cross‐feed with other microbiota species.^86^ As a consequence, *A. muciniphila* has been perceived to be beneficial for human health and is a potential probiotic.^87^, ^88^, ^89^ However, studies have shown that the enrichment of *Akkermansia* may play an important role in the progression of PD, as a significant trend effect for disease duration on increasing levels of *Akkermansia* was found.^25^, ^27^, ^33^, ^90^ Intriguingly, an increase in *Akkermansia* was also reported in multiple system atrophy, progressive supranuclear palsy,^33^ Alzheimer's disease, and multiple sclerosis,^52^ suggesting that this finding may not be specific to PD. Furthermore, multiple studies found *Akkermansia* to be relatively higher in oldest‐old adults and were positively associated with aging.^91^ Since PD is commonly seen in elderly people as shown in Table 1, the increased *Akkermansia* abundance may be associated with aging.

Our review of the literature is limited to a descriptive approach. Because these studies were disparate in their methodologies, examined heterogeneous populations, and reported on relative rather than absolute abundance, a meta‐analytic approach would be handicapped. PD medications may differentially alter components of the gut microbiome community, which may explain why the differences in some bacterial taxa varied unpredictably. Significant signals for COMT inhibitors, anticholinergics, and a borderline signal for carbidopa/levodopa have been found to be associated with the gut microbiome in PD patients.^37^ The genera *Phascolarctobacterium* and *Dorea* were negatively associated with levodopa equivalent doses, demonstrating that the microbiota might influence drug metabolism or that drugs might affect the microbiota.^19^ However, disease duration still significantly impacts microbial communities in PD patients, since a distinct correlation between numerous bacterial taxa and PD duration was maintained when untreated naїve samples were eliminated from the analysis.^36^ A limiting factor for this study is the inability to analyze the relationship between PD and microbiota without the interference of medications. Another limiting factor in interpreting results is the difficulty in comparing studies across different geographical regions. This is because participants' dietary habits, which have been shown to greatly impact gut microbial diversity, vary significantly from country to country. Overall, diets high in carbohydrates and saturated fats and low in fiber, commonly seen in the “Westernized” diet, are positively correlated with the SFACs producing the *Bacteroides* enterotype. Alternatively, the Asian diet is commonly lower in fat, which may promote a predominance of beneficial *Bifidobacteria* and a greater microbial diversity.^92^ Likewise, sex, exercise, and psychiatric symptoms could also be modulators of gut microbiota and could explain selected differences in bacterial taxa, which is hard to rule out when interpreting such diverse results.

## 5 CONCLUSION

**FIGURE 4:**
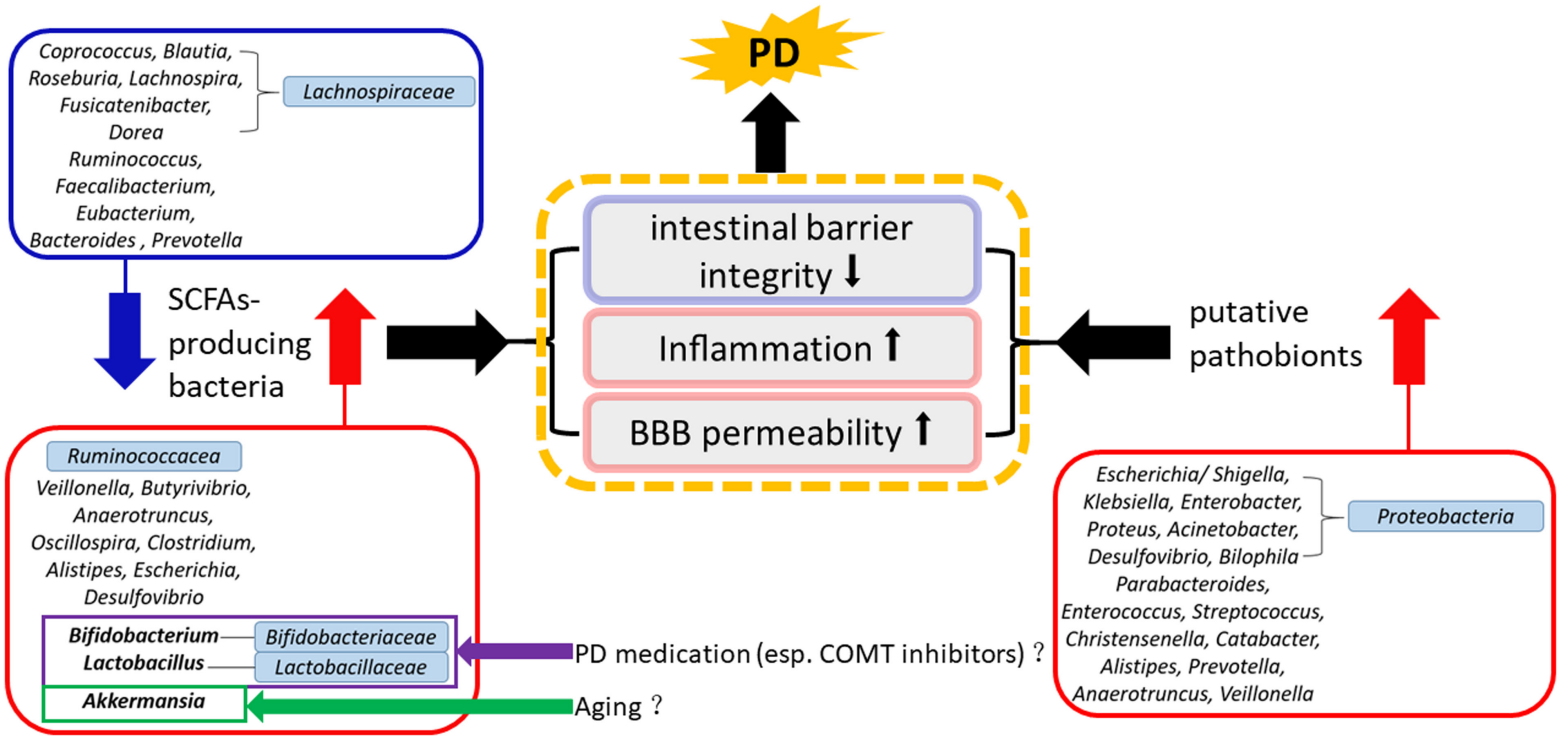
The possible gut bacteria (genus and family) alteration in the pathogenesis of Parkinson's disease. The blue box and arrow represent decreased abundance in PD patients compared to HCs. The red boxes and arrows represent increased abundance in PD patients compared to HCs. BBB, blood–brain barrier; COMT, catechol‐O‐methyltransferase; HC, healthy control; PD, Parkinson's disease; SCFAs, short‐chain fatty acids

Our review shows that the role of gut bacteria in association with PD may involve the alterations of bacteria with the capacity to produce SCFAs and an increase in putative pathobionts, which may work together to potentially impair the intestinal barrier and/or blood–brain barrier integrity, stimulating systemic and neural inflammation. SCFAs‐producing bacteria may reduce or increase outside of an “optimal range.” Considering that *Bifidobacterium*, *Lactobacillus*, and *Akkermansia* are beneficial for human health, the increased *Bifidobacterium* and *Lactobacillus* may be associated with PD medications, especially COMT inhibitors, while a high level of *Akkermansia* may be associated with aging. See Figure 4.

## CONFLICT OF INTEREST

The author declares no conflict of interest.

## Data Availability

The data that support the findings of this study are available from the corresponding author upon reasonable request.
